# Mouse chymase mast cell protease-4 facilitates blood feeding of *Aedes aegypti* (Diptera: Culicidae) mosquitoes

**DOI:** 10.1093/jme/tjaf137

**Published:** 2025-10-21

**Authors:** Zhiqiang Li, Xiaoyuan Kuang, Jiaxin Ling, Tao Shen, Ge Shan, Jiahong Wu

**Affiliations:** Guizhou Key Laboratory of Microbio and Infectious Disease Prevention and Control, Guizhou Medical University, Guiyang, China; Department of Immunology, College of Basic Medicine, Guizhou Medical University, Guiyang, China; Department of Medical Biochemistry and Microbiology, Uppsala University, Uppsala, Sweden; Guizhou Key Laboratory of Microbio and Infectious Disease Prevention and Control, Guizhou Medical University, Guiyang, China; Liupanshui Center for Disease Control and Prevention, Liupanshui, China; Department of Medical Biochemistry and Microbiology, Uppsala University, Uppsala, Sweden; Guizhou Key Laboratory of Microbio and Infectious Disease Prevention and Control, Guizhou Medical University, Guiyang, China; Department of Immunology, College of Basic Medicine, Guizhou Medical University, Guiyang, China; Guizhou Key Laboratory of Microbio and Infectious Disease Prevention and Control, Guizhou Medical University, Guiyang, China

**Keywords:** chymase mMCP-4, *Ae. aegypti*, blood feeding, skin microbiota

## Abstract

*Aedes aegypti* (Linnaeus) are rapidly spreading across the globe. Evidence suggests that a Type I hypersensitivity reaction, characterized by IgE-mediated mast cell degranulation, may enhance the blood-feeding behavior of *Ae. aegypti.* Chymases, the mast cell-specific proteases, may play a critical role in this process. To investigate the role of mouse chymase mast cell protease-4 (mMCP-4) on mosquito blood feeding, we incubated bone marrow-derived mast cells with serum from mice sensitized by female *Ae. aegypti* bites and subsequently challenged the cells with salivary gland proteins (SGPs) from female mosquito. And the degradation of SGPs by mMCP-4 was assessed. Then, the MCP-4 deficient mice were sensitized twice by *Ae. aegypti*, the first bite on day 0 and the second on day 3. Throughout these experiments, we recorded the total blood meal duration, probing time, and blood feeding of the mosquitoes and analyzed the cutaneous microbiota. We discovered that serum from sensitized mice enhanced mast cell degranulation and chymase release. And mMCP-4 degraded some SGPs, in particular, potentially cleaving the blood-feeding-related salivary protein D7. Mcpt-4 deficiency resulted in prolonged blood-feeding duration during the second exposure, without affecting initial probing behavior. Moreover, Mcpt-4-deficient mice exhibited a reduced proportion of mosquitoes achieving rapid engorgement. Skin microbiome profiling revealed that Mcpt-4 deficiency attenuated the bite-induced expansion of potentially harmful bacterial taxa, including the dominant genus *Corynebacterium* (Mycobacteriales: Corynebacteriaceae). These findings identify mMCP-4 as a critical mediator of mosquito blood-feeding behavior and a modulator of skin microbial ecology in response to *Ae. aegypti* bites.

## Introduction

Mast cells are a major immune cell population residing in the skin, characterized by cytoplasmic granules rich in preformed mediators, such as histamine, tryptases, and chymases. These mediators are essential for initiating rapid immune responses, particularly through promoting vasodilation, vascular permeability, and the recruitment of immune cells (Noto et al. 2021). Among these, mast cell protease-4 (mMCP-4) is a murine connective tissue-type chymase and monomeric serine protease, sharing substrate specificity and tissue distribution with human chymase (Andersson et al. 2008). And it has been implicated in various physiological and pathological processes, such as atopic dermatitis, wound healing, and tissue remodeling (Svanberg et al. 2020, Mogren et al. 2022). Functionally, chymase increases vascular permeability by degrading intercellular junction proteins (Bankova et al. 2014, Pejler 2020), and elevated levels have been proposed as biomarkers for severe dengue infection, a mosquito-borne disease (St John et al. 2013, Durbin 2019).

*Aedes aegypti* (L.), the invasive yellow fever mosquito, poses a significant public health challenge due to its role in transmitting viral pathogens and inducing allergic skin reactions (Ahebwa et al. 2023). During blood feeding, female mosquitoes inject a complex mixture of salivary proteins into the host skin. While the full spectrum of their functions remains incompletely characterized, several of these proteins exhibit anti-inflammatory and anesthetic properties that facilitate efficient blood meal acquisition by shortening feeding time (Cantillo et al. 2023). Some salivary proteins induce the production of IgE antibodies in the host, leading to Type I hypersensitivity reaction that further alters the host immune environment (Cantillo et al. 2023).

Type I hypersensitivity is an IgE-mediated allergic reaction that plays a critical role in immune responses to environmental allergens, including those introduced during mosquito bites. During blood feeding, salivary proteins from *Ae. aegypti* initiate antigen capture by dendritic cells and subsequent presentation to naïve T cells. This antigen presentation activates B cells, leading to the production of allergen-specific IgE antibodies, a process that typically develops over several days. Circulating IgE then binds to high-affinity FcεRI receptors expressed on the surface of skin-resident mast cells. Upon subsequent exposure to mosquito salivary allergens, these sensitized mast cells undergo degranulation, releasing a suite of proinflammatory mediators, including mast cell-specific serine proteases such as chymases.

Type I hypersensitivity has been shown to enhance *Ae. aegypti* blood feeding (Conway 2021), likely through mechanisms that promote local vasodilation and vascular permeability (­Martin-Martin et al. 2022). Notably, the enzymatic activity of human chymase is enhanced by specific salivary proteins from *Aedes albopictus*, such as 34k2 and adenosine deaminase (Li et al. 2022). This suggests that mosquito saliva and host mast cells may interact in complex and potentially reciprocal ways. Moreover, there is growing evidence that connective tissue-type chymases play a protective role by degrading anticoagulant proteins from ticks, leeches, and mosquitoes (Fu et al. 2021). In turn, these ectoparasites have evolved protease inhibitors that specifically block chymase and tryptase activity, underscoring an evolutionary arms race at the host–vector interface (Fu et al. 2021). However, the precise functional consequences of chymase during mosquito feeding remain poorly understood.

The skin microbiota plays a critical role in modulating skin immunity, and dysbiosis of these microbial communities can lead to cutaneous inflammatory and autoimmune diseases (Belkaid and Segre 2014, Harris-Tryon and Grice 2022). Mast cells, which reside abundantly in the skin, are key mediators in these immune responses and maintain a close interplay with the microbiota. Under physiological conditions, mast cell-derived mediators support epithelial barrier integrity and coordinate mucosal immune responses (Papa et al. 2025). However, in pathological contexts, aberrant mast cell activation can compromise barrier function, promoting an environment favorable to the proliferation of pathogenic bacteria (Papa et al. 2025). Beyond their immunological role, skin microbiota significantly influences interactions between hosts and mosquitoes by producing volatile organic compounds that affect individual attractiveness to these insects (Michalet et al. 2019). During blood feeding, mosquitoes inject salivary proteins into the dermis, triggering local immune responses, such as inflammation, vasodilation, and pruritus (Henrique et al. 2019). These responses are further shaped by the skin’s microbial composition, underscoring a complex and reciprocal relationship between the skin microbiota, mast cell-mediated immunity, and mosquito-host interactions.

Given these observations, we hypothesize that chymase mMCP-4 could play significant role in modulating mosquito blood feeding and influencing skin microbiota. To verify this, bone marrow-derived mast cells (BMMCs) were sensitized with serum from mice previously exposed to *Ae. aegypti* bites and stimulated with mosquito salivary gland proteins (SGPs) to assess degranulation responses and chymase release. To directly evaluate the proteolytic potential of mast cell proteases against mosquito salivary components, SGPs were incubated with recombinant mouse chymase MCP-4 or tryptase MCP-6. SDS-PAGE and mass spectrometry analyses revealed that MCP-4, but not MCP-6, selectively cleaved specific SGPs, including members of the D7 protein family, key molecules known to facilitate blood feeding. Additionally, using conditional Mcpt-4 knockout mice, we assessed the in vivo consequences of mMCP-4 deficiency on mosquito feeding behavior and cutaneous microbial composition following bites. Collectively, these experiments aimed to elucidate the dual role of mMCP-4 at the host–vector interface: both in regulating mosquito access to blood and in shaping post-bite skin microbiota.

## Materials and Methods

### Mosquitoes and Soluble Salivary Gland Proteins

Female *Ae. aegypti* were fed with sterile fresh 10% sugar and reared at 25 ± 1 °C and a light–dark 14:10 photoperiod. To get soluble salivary gland proteins, a total of 20 female *Ae. aegypti* were placed in a cell culture dish then held at in −20 °C for 1 min, and after that salivary glands were dissected out with 2 fine-tipped tweezers. The pooled glands were resuspended in 400 μl of sterile phosphate-buffered saline (PBS) then disrupted using an ultrasonic disintegrator (JY92-IIN, Leibao, China), and the homogenate was centrifuged at 8,000 rpm for 5 min. The concentration of protein was determined by using BCA Protein Assay Kit (ThermoFisher, #23250). The supernatant was kept in −80 °C until used.

### BMMCs: Preparation, Culture, and In Vitro Challenge

BMMCs were cultured and differentiated from the isolated cells in femur and tibia of C57BL/6J mice as described (Li et al. 2019). Cells were washed twice with PBS and cultured with the completed RPMI1640 medium supplemented with 10% fetal bovine serum (Gibco, A5256701), 1% Penicillin/Streptomycin (Gibco, 10378016), 1% mercaptoethanol (Gibco, #21985023), 5% nonessential amino acid (Gibco, #11140050), 20 ng/ml of recombinant murine SCF (Peprotech, #250-03), and 20 ng/ml of IL-3 (Peprotech, #213-13) at conditions of 37 °C and 5% CO_2_ in a cell incubator. After 3 wk, mast cells were verified with May–Grünwald/Giemsa staining.

The BMMCs were washed 3 times with PBS and seeded in 24 well plates at 0.5 × 10^6^ cells per 0.5 ml in serum-free Hanks’ balanced salt solution medium and then incubated 30 min with 0.05 ml pooled serum extracted from blood of 5 mice with or without bites of *Ae. aegypti* to generate IgE—bound mast cells. After washing 3 times, the cells were stimulated with soluble mosquito SGPs at final concentrations of 1, 10, or 100 ng/ml for 6 h. Lipopolysaccharide was used as a positive control for mast cell activation. Supernatants were collected and stored at −80 °C for subsequent biochemical analyses.

### Measurement of β-Hexosaminidase and Chymase Activities

Mosquito bites are known to induce the production of IgE antibody, which binds to Fc receptor on mast cells. By capturing mosquito SGPs, they boost the degranulation of mast cell (Vander Does et al. 2022). However, it remains unclear whether the level of chymase is elevated following this process. To address this, we incubated the BMMCs with the serum extracted from the blood of *Ae. aegypti*-bitten mice (3 d post the bites) or naïve (unbitten) mice. We then assessed the activities of mast cell degranulation marker proteins, including β-hexosaminidase and chymase. For each serum group, a 0 ng SGP condition (serum incubation without SGP stimulation) was included as a baseline comparison. Comparative analyses were conducted between the sensitized and non-sensitized serum groups.

The BMMCs were prepared as described previously, then incubated with the serum collected from the blood of *Ae. aegypti bitten* mice. After 30 min, cells were washed 3 times with PBS and then challenged with *Ae. aegypti* SGPs for 6 h. The activity of mast cell degranulation marker proteins β-hexosaminidase and chymase were determined. β-hexosaminidase is a mediator stored in mast cells, playing a key role in their activation and degranulation during immune responses. The measurement of β-hexosaminidase is frequently used for determining the activation state of MC.

For the measurement of β-hexosaminidase activity, we followed the protocol as previously described (Li et al. 2019). Briefly, 80 μl of cell supernatants were incubated with 20 μl of 5 mM p-nitrophenyl N-acetyl β-D-glucosamine (Absin, #abs42136928) dissolved in citrate buffer for 1 h at ambient temperature. After adding 200 μl 0.05 M sodium carbonate reaction buffer, the absorption was measured at 405 nm post-treatment 0 and 4 h and the difference of the optical density (OD) value for absorbance was calculated. Data from 2 independent experiments, each performed with 3 technical replicates, were pooled (*n* = 6) for statistical analysis.

For the chymase assay, 80 μl of cell supernatants were incubated with 20 μl of 2 mM substrate, L-1595 (Chromogenic, Sweden), at room temperature for overnight. The absorbance at 405 nm was determined immediately post 0 and 24 h after reaction, and the difference in optical density was calculated. Data from 2 independent experiments, each with 3 technical replicates, were pooled for statistical analysis (*n* = 6 per condition).

### Chromogenic Substrate and SGPs Degradation Assays

To verify the enzymatic activity of recombinant mMCP-4 (Cat# RPC25130, Biomatik), 10 ng of protein was dissolved in 100 µl of sterile PBS (0.15 M NaCl) in the presence or absence of 10 ng of the chymase inhibitor Fulacimstat (#HY-109059, MedChemExpress). At room temperature, 20 µl of the chromogenic substrate L-1595 (2.5 mM) was added. Optical density (OD) was recorded at 405 nm at 0, 10, 20, 30, and 60 min using a microplate reader.

To evaluate the enzymatic degradation of mosquito SGPs by mouse mast cell proteases, 20 μg of SGPs were incubated with either 0.01 μg of recombinant mouse tryptase MCP-6 (Cat# 3736-SE, R&D Systems) or 0.01 μg of recombinant mouse chymase MCP-4 Reactions were carried out at room temperature (∼22 °C) for approximately 18 h to allow for proteolytic cleavage. As a control, SGPs were incubated under identical conditions without the addition of proteases. Following incubation, samples were subjected to SDS-PAGE, and proteins were visualized by Coomassie Brilliant Blue staining to assess degradation profiles.

### Coomassie Blue Staining of SDS-PAGE Gels

Following electrophoresis, SDS-PAGE gels containing SGP samples were stained using standard Coomassie blue procedures. Gels were incubated overnight in a staining solution containing 0.1% Coomassie Brilliant Blue R-250, 50% methanol, and 10% acetic acid. After staining, gels were destained using a buffer composed of 10% acetic acid, 40% ethanol, and 50% sterile distilled water. The destaining process was carried out for 40 min with 3 to 4 buffer changes to remove excess dye. Gel images were captured using the imaging system.

### Protein Identification by LC-MS/MS

Protein identification was performed, as described in Li et al. (2022). In brief, protein band of interest (marked with arrow in Supplementary Table S1) were excised from SDS-PAGE gel, destained with 50% acetonitrile in 100 mM ammonium bicarbonate (pH 8.0), reduced with 10 mM dithiothreitol, and alkylated with 60 mM iodoacetic acid. After drying, gel pieces were digested overnight at 37 °C with trypsin (15 ng/μl). Peptides were extracted using 0.1% formic acid in acetonitrile and analyzed by Liquid Chromatography–Tandem Mass Spectrometry (LC-MS/MS) using a Q Exactive Orbitrap mass spectrometer (Thermo Scientific). Separation was achieved on a C18 column with a 100-min gradient. MS data were acquired in data-dependent mode and searched using Proteome Discoverer v2.2 against the *Ae. aegypti* UniProt database. Peptide-­spectrum matches were filtered based on delta Cn ≥0.05, and ­contaminant/reverse hits were excluded from final results.

### Mice

Ten- to 12-wk-old wide type C57BL/6J mice were anesthetized with 75 mg/kg of ketamine or (and) 10 mg/kg of xylazine by intraperitoneal injection. Each mouse was immobilized with a mouse fixator and exposed for approximately 3 h to 100 female mosquitoes starved for 24 h in a cage that was sealed with an 8,000 cm^3^ cotton net. Three days later, mice were euthanized using CO_2_ gas, blood was collected, and serum was extracted and stored at −80 °C for further analysis.

The induction of knockout of chymase mMCP-4 (Mcpt-4, Gene ID: 17227) in C57BL/6J mice was established using tamoxifen-inducible Cre-loxP system (Hayashi and McMahon 2002). The mice were kept in the Department of Immunology, College of Basic Medicine, Guizhou Medical University. Permission (No. 2500104) for experiments with mice was granted by Guizhou Medical University’s Experimental Animal Center. All experimental female mice were age-matched littermates and were treated humanely and housed in an enriched environment. Although different genotypes were housed in separate cages, this practice is commonly employed in skin disease models to prevent microbiota cross-contamination while preserving environmental consistency.

### Generation and Validation of Conditional Mcpt-4 Knockout Mice

As previously described (Li et al. 2025), conditional knockout mice for Mcpt-4 were generated on a C57BL/6J background using a floxed allele with loxP sites inserted into introns 1 and 4. These Mcpt-4^flox/flox^ mice were crossed with CAGGCre-ER mice, allowing for tamoxifen-inducible Cre-mediated deletion. Cre genotyping was confirmed by PCR, and deletion of Mcpt-4 was induced by intraperitoneal injection of tamoxifen (20 mg/kg/day for 5 d). Total RNA was extracted from tail tissue using TRIzol (Thermo Fisher, #15596026), followed by cDNA synthesis and qPCR with gene-specific primers to confirm Mcpt-4 deletion. GAPDH was used as a reference gene. Full methodological details are available in our prior publication (Li et al. 2025).

### Probing Time, Blood Feeding Time, and Blood-Feeding Success Rate

As described in Martin-Martin et al. (2022), probing time is when mosquito intradermally search for blood vessel and is defined as the interval from the initial insertion of the mouthparts into the skin until visualization of first traces of blood in the midgut. Feeding time is the interval between the end of probing time and the time instantly after a fully engorged mosquito pulls out the proboscis and flies away. Blood-feeding success rate is defined as the percentage of mosquitoes that successfully accomplish blood feeding with a full engorgement after probing. Here, littermate and age-matched Mcpt-4^flox/flox^ control and Mcpt-4^flox/flox/Cre^ female mice (n≧3 in each group) were sensitized to bites by female *Ae. aegypti* on experiment days 0 and 3. Mice were anesthetized using 75 mg/kg of ketamine or (and) 10 mg/kg of xylazine and were immobilized with a mouse fixator and exposed to 60 female mosquitoes in a cage sealed with an 8,000 cm^3^ cotton net. These female mosquitoes are 3- to 7-d-old and had been deprived for 24 h. The number of mosquitoes taking blood within approximately 2 h were monitored.

As not all mosquitoes initiated feeding simultaneously, each mosquito was monitored individually to accurately capture its probing initiation and blood feeding completion times. Five trained observers were assigned to monitor the mosquitoes, with each observer covering a designated portion of the cage. Observers continuously recorded the probing and blood feeding times of individual mosquitoes in real time using stopwatches. The number of mosquitoes that achieved full engorgement was quantified. Blood feeding rates during the first and second biting events was assessed. The success rate of blood feeding mosquitoes with full engorgement within 90, 120, 150, or 180 s during both the first and second biting events were compared. Mosquito feeding assays were performed twice (experiments #1 and #2), probing time and feeding time in experiment #1 were recorded, success rates were calculated from experiments #1 and #2.

### 16S rRNA Gene Sequencing and Microbiome Analysis

Mosquito bites can shape the skin immune microenvironment, and the immune system has very strong connection with skin microbiota (Belkaid and Segre 2014, Harris-Tryon and Grice 2022). To investigate whether mMCP-4 affects skin microbiota upon sensitization by *Ae. aegypti bites*, we collected the cutaneous bacteria from mice in experiment #2 on day 2, before the second biting event, for microbiota analysis.

Total DNA was isolated from skin surface collected from experimental mice using the DNeasy PowerSoil Pro Kit (­QIAGEN, United States) according to the supplier’s protocol. The 16S rRNA V3–V4 hypervariable regions of cutaneous bacteria were amplified with the extracted genomic DNA using primers 338 Forward (5′-ACT CCT ACG GGA GGC AGC AG-3′) and 806 Reverse (5′-GGA CTA CHV GGG TWT CTA AT-3′). The PCR products were examined by gel electrophoresis and purified with the AxyPrep DNA Gel Extraction Kit (Axygen Biosciences, Axygen, United States). Sequence libraries were generated using the NEXTFLEX Rapid DNA-Seq Kit. Sequencing was performed using the Illumina MiSeq PE300/NovaSeq PE250 platform (Illumina, San Diego, United States) according to the manufacturer’s instructions (Majorbio, Shanghai, China). Quality of the original sequence data was assessed using fastp (https://github.com/OpenGene/fastp, version 0.20.0) and spliced using FLASH (https://github.com/OpenGene/fastp, version 1.2.7). Operational taxonomic units (OTUs) were clustered based on a 97% similarity threshold by UPARSE (version 7.1). The analysis of taxonomy for each sequence was performed by comparing the Ribosomal Database Project (RDP) Classifier against the 16S rRNA database. The disruption of bacteria composition was evaluated with microbial dysbiosis index (MDI, MDI=log_10_ [(total abundance in genera increased in disease group)/(total abundance in genera decreased in disease group)]). The α-diversity of bacteria reflecting the richness (number of species) and evenness (distribution of species) of bacterial populations was calculated based on the OTUs using the coverage index. The β-diversity of the bacteria describing the diversity between different microbial communities was estimated with principal component analysis of Bray–Curtis distance. Differences in bacteria based on relative abundance on the genus levels were analyzed using the Kruskai–Wallis H test to compare 4 groups. The data were analyzed through the majorbio cloud platform (cloud.majorbio.com). The datasets presented can be found in online repositories (NCBI, PRJNA1149461).

### Statistical Analysis

**Fig. 1.**
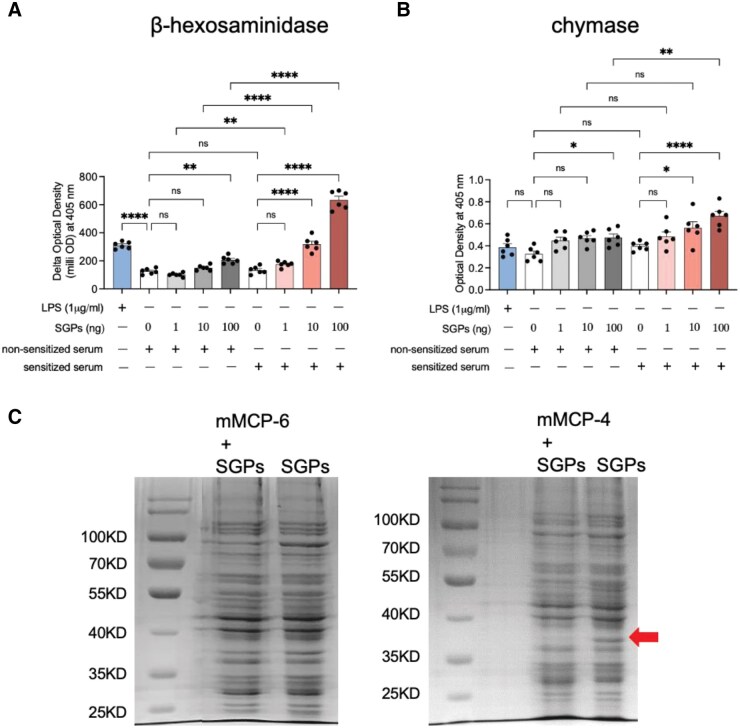
Sensitized serum enhances mast cell degranulation and chymase production, and chyamse mMCP-4 degrades some *Aedes aegypti* salivary proteins (SGPs). A) β-hexosaminidase or B) chymase enzyme activity was measured in BMMCs incubated with serum from mosquito-sensitized or naïve mice and stimulated with SGPs at the indicated concentrations (0, 1, 10, or 100 ng/ml). Lipopolysaccharide (1 μg/ml) was used as a positive control. “+” or “−” indicates the presence or absence of non-sensitized serum, sensitized serum, and SGPs for each condition. Data are presented as means ± SEM from 2 independent experiments with 3 technical replicates each (*n* = 6 per condition). C) 20 μg of the extracted SGPs were incubated with 0.01 μg of recombinant mouse tryptase MCP-6 or chymase MCP-4 for overnight (approximately 18 h). SDS-page gels were stained with coomassie blue. Proteins marked with arrow were identified by liquid chromatograph mass spectrometer. Statistical analysis was performed using ordinary 1-way ANOVA followed by Sidak’s multiple comparisons test for preselected pairs. *P < 0.05; **P < 0.01; ****P < 0.0001; ns, not significant.

**Fig. 2.**
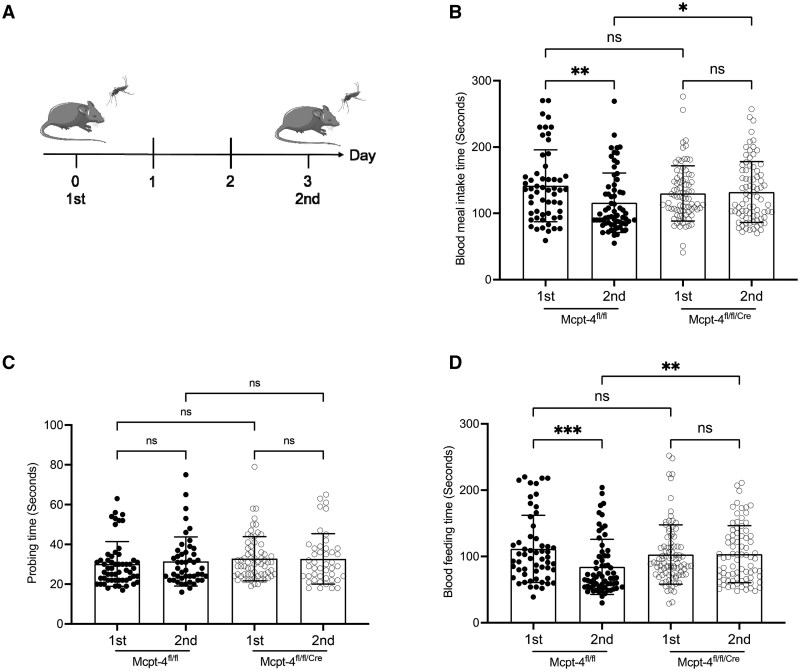
The Mcpt-4 deficiency in mice prolonged blood feeding time of *Ae. aegypti* mosquito. A) Experimental timeline using age matched Mcpt-4^fl/fl^ control and Mcpt-4^fl/fl/Cre^ female mice for mosquitoes (*n* = 60) exposure on days 0 and 3. The entire time of blood meal intake B), the probing time C) and blood feeding time D) of mosquitoes with partial and full engorgement were statistically analyzed. Each dot represents an individual mosquito. Data are presented as means ± SD. Line symbol indicates the mean, and statistical significance was assessed using Kruskal–Wallis test; ns, not significant, *P < 0.05, **P < 0.01, ***P < 0.001. Figure 2A was created using BioIcons open-source icons. Icon used in the mice figure are copyrighted under license CC-BY 3.0. Mosquito icon is licensed under CC-BY 4.0.

**Fig. 3.**
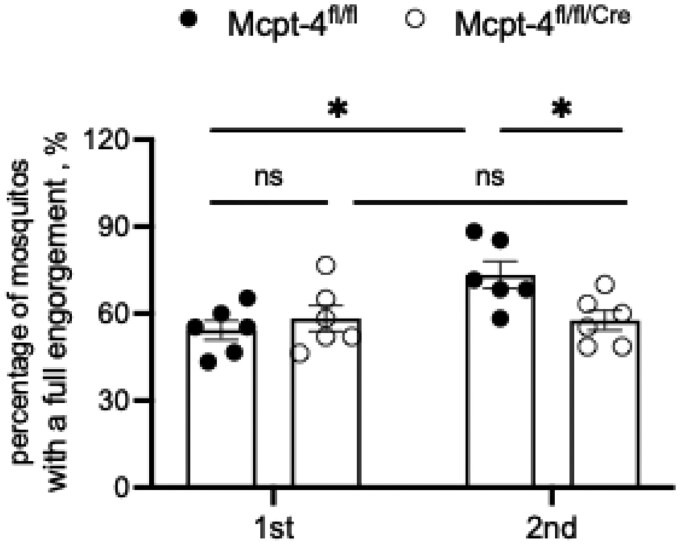
The lack of Mcpt-4 reduced the blood feeding success rate at the second biting of *Ae. aegypti*. All experimental mice (*n* = 6 for each group, pooled from 2 independent experiments) were experienced twice exposures to the deprived female mosquitos, the percentage of *Ae. aegypti* numbers finished blood feeding with successfully and fully engorged post probing at the first and second bites were statistically evaluated. Each dot represents a mouse. Data are shown as mean ± SE, and statistical analysis was performed using 1-way ANOVA followed by Sidak’s multiple ­comparisons test for preselected pairs. ns, no significance, *P < 0.05.

**Fig. 4.**
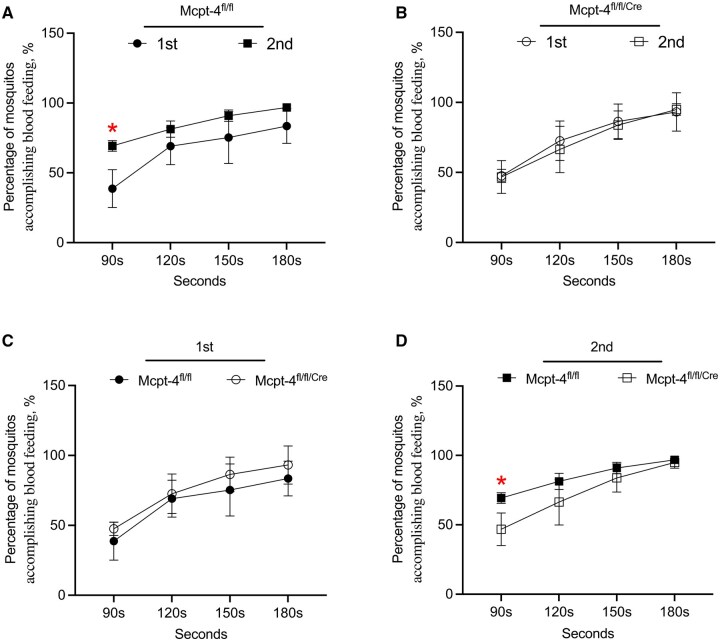
The lack of Mcpt-4 slowed the onset of the blood feeding on sensitized mice. All experimental mice (*n* ≧ 3 for each group) experienced exposures to the deprived female mosquitoes (*n* = 60), the percentage of *Ae. aegypti* obtained full engorgement post blood feeding at 90, 120, 150, or 180 s in the first and second bites were statistically evaluated A) to D). Data are shown as mean ± SD, and statistical analysis was performed using 1-way ANOVA followed by Sidak’s multiple comparisons test for preselected pairs. *P < 0.05.

For Fig. 1, multiple technical replicates were generated within a single experiment. For Fig. 3, 3 mice per group were used in 2 independent experiments (*n* = 6 per group). For Fig. 4, data were collected from a single experiment. Replicates were treated as independent observations. Normality was assessed based on visual inspection of data distribution plots. One-way ANOVA followed by Sidak’s multiple comparisons test was used for Figs. 1, 3, and 4. The Kruskal–Wallis test was used for Fig. 2 to accommodate nonparametric data distributions. *P*-values < 0.05 were considered statistically significant.

## Results

### Mast Cell Degranulation and mMCP-4 Chymase Release Were Enhanced by Ae. aegypti Sensitization, and mMCP-4 Selectively Degraded SGPs

To assess the potential role of chymase in host–mosquito interactions, BMMCs were stimulated with varying concentrations of *Ae. aegypti* SGPs, either in the presence or absence of serum from mosquito-sensitized mice. As shown in Fig. 1A, exposure to 100 ng of SGPs alone induced detectable mast cell degranulation, as measured by β-hexosaminidase release (*t* = 4.195, df = 45, *P* = 0.0014). This finding is further supported by the Supplementary Data (*t* = 8.473, df = 15, *P* < 0.0001) (Supplementary Fig. S1), demonstrating that *Ae. aegypti* SGPs can independently trigger mast cell degranulation without prior serum sensitization. Quantitative analysis revealed that the presence of sensitized serum significantly elevated β-hexosaminidase (*t* = 22.03, df = 45, *P* < 0.0001) and chymase (*t* = 3.964, df = 45, *P* = 0.0029) activities, particularly at the 100 ng SGP concentration (Fig. 1A and 1B). Which suggests that mast cells pre-incubated with serum from sensitized mice exhibited significantly enhanced degranulation compared to those treated with non-sensitized serum, supporting a role for mast cell sensitization in promoting an amplified degranulation response to SGPs.

To confirm the enzymatic activity of the recombinant mMCP-4 used in our assays, we first performed a chromogenic substrate assay, as described in Li et al. (2022). Recombinant mMCP-4 showed robust cleavage of the chymase substrate L-1595, whereas activity was completely abolished in the presence of the specific inhibitor Fulacimstat (Supplementary Fig. S2). Then *Ae. aegypti* SGPs were incubated with recombinant mouse mast cell proteases mMCP-6 (tryptase) or mMCP-4 (chymase) to assess differential proteolytic activity. SDS-PAGE followed by coomassie blue staining (left and right panels) revealed no significant degradation in the presence of mMCP-6, while mMCP-4 treatment resulted in a visible loss of a prominent ∼36 kDa protein band (red arrow), indicating selective cleavage (Fig. 1C). Mass spectrometry analysis of the degraded band identified several proteins with high confidence, including long-form salivary protein D7L1 (P18153) (Supplementary Table S1), which has been shown to affect probing time, supporting its potential role in efficient blood feeding. Supplementary peptide mapping further revealed 5 predicted chymase cleavage sites within the D7L1 protein sequence, characterized by aromatic amino acids (F, W, Y) at the P1 position and acidic residues at P2’, consistent with chymase substrate specificity (Supplementary Fig. S3). These findings suggest that chymase mMCP-4 may interfere with mosquito blood feeding by degrading critical salivary components such as D7L1.

### Mcpt-4 Deficiency in Mice Resulted in Prolonged Blood Feeding Time for Ae. aegypti in Sensitized Mice

To investigate the role of chymase MCP-4 in mosquito blood feeding, we conducted an experiment using MCP-4 deficient mice as illustrated in Fig. 2A. The duration of the second blood meal was significantly shorter compared to the first in mosquitoes fed on Mcpt-4^fl/fl^ control mice (*Z* = 3.099, *P* = 0.0078). However, mosquitoes fed on Mcpt-4-deficient (Mcpt-4^fl/fl/Cre^) mice showed no significant difference in blood feeding duration between the first and second exposures (Fig. 2B). While Mcpt-4 deficiency did not affect the total intake time during the initial feeding, it significantly prolonged the duration of the second blood meal (*Z* = 2.510, *P* = 0.0483; Fig. 2B). To further determine the specific stage of blood meal intake affected by Mcpt-4, we separately analyzed the probing time and blood feeding time. There were no significant differences in probing time among any groups (Fig. 2C). Conversely, blood feeding time during the second exposure was significantly increased in Mcpt-4-deficient mice (*Z* = 3.174, *P* = 0.006; Fig. 2D), mirroring the overall meal duration results. These observations suggest that sensitization to mosquito bites typically shortens subsequent blood feeding durations, and this effect is disrupted by Mcpt-4 deficiency.

### The Lack of Mcpt-4 Reduced the Blood Feeding Success Rate at the Second Biting

Next, we evaluated the effect of chymase MCP-4 on the blood feeding success. As shown in Fig. 3, there was no significant difference in the blood-feeding success rate at the first bite between Mcpt-4^fl/fl^ control mice and Mcpt-4^fl/fl/Cre^ mice. Similarly, Mcpt-4^fl/fl/Cre^ mice showed no significant change in feeding success between the first and second exposures. In contrast, Mcpt-4^fl/fl^ control mice exhibited a significant increase in blood-feeding success during the second bite compared to the first (*t* = 3.348, df = 20, *P* = 0.0128). This enhancement in feeding success was notably diminished in Mcpt-4-deficient mice (*t* = 2.762, df = 20, *P* = 0.0356), indicating that Mcpt-4 plays a role in promoting efficient blood feeding at the second biting.

### The Lack of Mcpt-4 Slowed the Onset of the Blood Feeding on Sensitized Mice

Next, we compared the proportion of mosquitoes reaching full engorgement at various time points at the first and second blood-feeding events. In Mcpt-4^fl/fl^ control mice, a significantly greater percentage of mosquitoes reached full engorgement during the second exposure compared to the first, with a statistically significant difference observed at the 90-s mark (*t* = 3,174, df = 20, *P* = 0.019; Fig. 4A). In contrast, no significant difference was observed between the first and second exposures in Mcpt-4^fl/fl/Cre^ (Mcpt-4-deficient) mice (Fig. 4B), indicating a lack of feeding enhancement upon sensitization. Furthermore, during the first exposure, there was no significant difference in the rate of mosquito engorgement between the 2 genotypes (Fig. 4C). However, during the second exposure, the percentage of mosquitoes that achieved full engorgement at 90 s was significantly reduced in the Mcpt-4-deficient group compared to controls (*t* = 2.183, df = 20, *P* = 0.038; Fig. 4D). These findings indicate that Mcpt-4 contributes to the accelerated onset of mosquito feeding in sensitized hosts and that its absence disrupts this facilitation, delaying effective blood meal acquisition by *Ae. aegypti*.

### Mcpt-4 Deficiency Modified the Composition of Cutaneous Microbiota in Ae. aegypti Bitten Mice

**Fig. 5.**
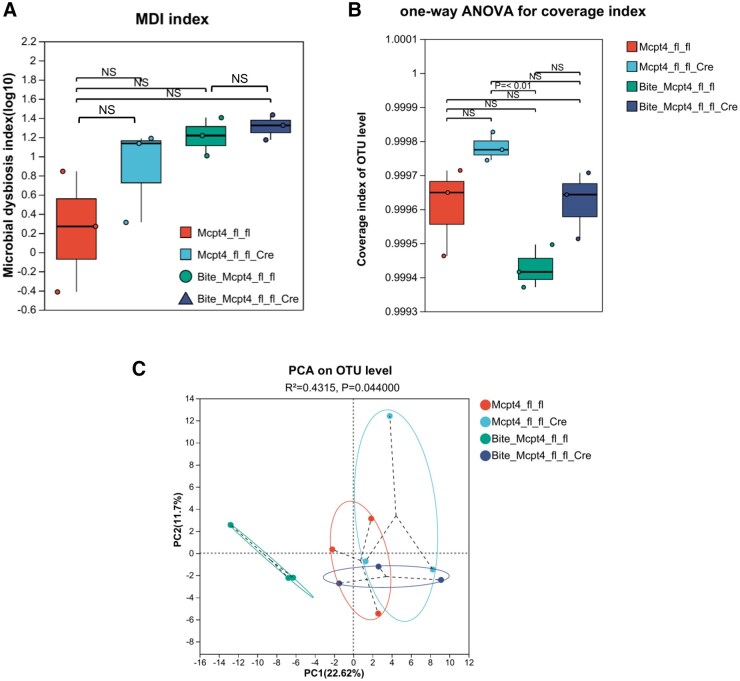
The Mcpt-4 deficiency altered the α-diversity and β-diversity of cutaneous microbiota in *Ae. aegypti* bitten mice. The microbial dysbiosis index (MDI) A), α-diversity represented by the coverage index B) and β-diversity on OTU C) level among Mcpt-4^fl/fl^, Mcpt-4^fl/fl/Cre^, Bite+ Mcpt-4^fl/fl^, and Bite+Mcpt-4^fl/fl/Cre^ groups (3 mice for each group) were compared. NS, no significance.

**Fig. 6.**
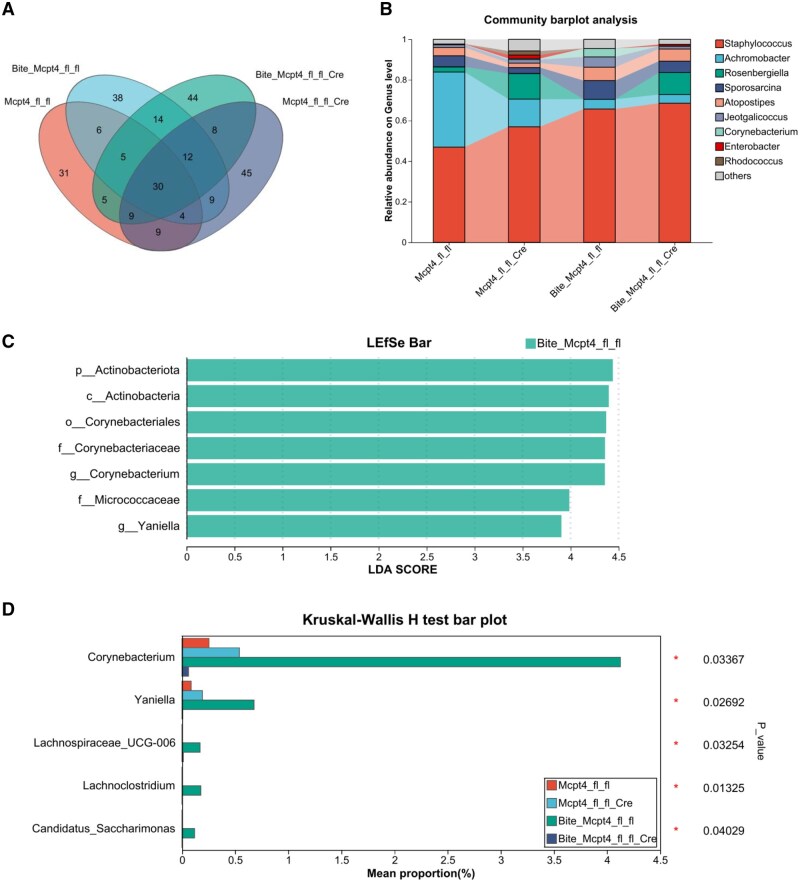
The Mcpt-4 deficiency caused the significant reduction of potential harmful bacteria in mosquito bitten mice. A) The bacteria on genus level were counted among Mcpt-4^fl/fl^, Mcpt-4^fl/fl/Cre^, Bite+Mcpt-4^fl/fl^, and Bite+Mcpt-4^fl/fl/Cre^ groups (3 mice for each group). The percent of community abundance of cutaneous bacterial on genus level was shown in B). C) The linear discriminant analysis of effect size was calculated, and D) the bacteria on genus level were statistically analyzed using Kruskal–Wallis *H* test and bacteria with significant difference were shown. Significant difference presented as * *P* < 0.05.

To assess the impact of Mcpt-4 on skin microbiota in response to *Ae. aegypti* bites, we compared microbial community metrics between Mcpt-4^fl/fl^ control and Mcpt-4-deficient (Mcpt-4^fl/fl/Cre^) mice. As shown in Fig. 5A, there were no significant differences in the MDI across groups, indicating a comparable global imbalance of microbial communities. However, mosquito bite sensitization significantly altered both α-diversity (as measured by coverage index) and β-diversity in Mcpt-4^fl/fl^ control mice, but not in Mcpt-4-deficient mice, suggesting that Mcpt-4 plays a role in modulating the microbial diversity upon mosquito challenge (Fig. 5B and C). Genus-level taxonomic analysis revealed that 30 bacterial genera were shared among all 4 groups (Mcpt-4^fl/fl^, Mcpt-4^fl/fl/Cre^, Bite+Mcpt-4^fl/fl^, and Bite+Mcpt-4^fl/fl/Cre^), while unique genera counts were 31, 45, 38, and 44, respectively for each group (Fig. 6A). This pattern indicates that mosquito bites and Mcpt-4 deficiency each influence the bacterial landscape independently. Further analysis showed that Mcpt-4 deficiency resulted in pronounced shifts in genus-level relative abundance in mosquito-bitten mice (Fig. 6B). Linear discriminant analysis effect size (LEfSe) identified 7 dominant genera unique to the Bite+Mcpt-4^fl/fl^ group that significantly contributed to intergroup variation (Fig. 6C). Five genera exhibited statistically significant changes in abundance: *Corynebacterium* (*P* = 0.03367), *Yaniella* (Micrococcales: Micrococcaceae) (*P* = 0.02692)*, Lachnospiraceae_UCG-006* (Eubacteriales: Lachnospiraceae) (*P* = 0.03254)*, Lachnoclostridium* (Eubacteriales: Lachnospiraceae) (*P* = 0.01325), and *Candidatus_Saccharimonas* (Saccharimonadales: Saccharimonadaceae) (*P* = 0.04029) (Fig. 6D). These taxa are considered potentially harmful and are associated with skin inflammation and dysbiosis, highlighting a potential immunomodulatory role of Mcpt-4 in shaping host–microbiota interactions following *Ae. aegypti* exposure.

## Discussion

Mosquitoes are rapidly spreading worldwide, causing a serious public health concern. In addition to causing irritating cutaneous allergic reaction, they can transmit various viruses and parasites. During probing and blood intake, the salivary proteins (SPs) are injected into the host skin where many types of immune cells reside, including the abundantly located mast cells. Some salivary proteins can directly activate mast cells (MCs), such as adenosine deaminase and 34k2 from Ae. albopictus (Li et al. 2022). Additionally, some SPs like Aed a 1-3 can trigger type I hypersensitivity, leading IgE mediated MCs degranulation (Cantillo et al. 2023). Although the levels of IgE is very low in the blood, this cytotropic antibody binds directly to MCs, allowing them to capture antigens. MCs express various receptors, including IgE receptors and Toll-like receptors, enabling activation through both IgE dependent and IgE-independent ways. Based on this, we designed an experiment where bone marrow derived MCs were incubated with the serum from mice sensitized by mosquito bites, while non sensitized serum was used as a control. Our data showed that SGPs alone could induce MCs degranulation, and this effect was further enhanced by sensitized serum, leading to increased release of β-hexosaminidase and chymase. Furthermore, type I hypersensitivity has been found to promote the blood feeding for *Ae. aegypti* (Conway 2021).These suggest that mosquito sensitization primes mast cells for a heightened degranulation response upon subsequent exposure to mosquito salivary antigens. Given the central role of mast cells in IgE-mediated type I hypersensitivity responses, these results reveal that chymase release during mast cell degranulation may facilitate mosquito blood feeding by modulating the local immune and vascular environment.

MCs are the key immune cells involved in allergic reactions, and they preferentially locate at sites that interface with the external environment. Within skin tissues, MCs are found in close proximity to nerves, blood vessels, hair follicles, adipose tissue, and muscle tissue. In humans, chymase is produced by MCs that express both chymase and tryptase. In mice, the chymase-related genes include Mcpt-1, Mcpt-4, and Mcpt-9, while tryptase-related genes include Mcpt-6 and Mcpt-7. It is worth noting that BMMCs, which were used in activation assays in this study, do not express high levels of mMCP-4 under typical IL-3 culture conditions. Instead, they predominantly express mMCP-5 (now classified as a chymotrypsin-like elastase), mMCP-6 (tryptase), and carboxypeptidase A3 (Akula et al. 2020, Berglund et al. 2020). Although mMCP-2 is structurally related to chymases, it exhibits minimal enzymatic activity and is no longer considered a functionally active protease (Pemberton et al. 2003). These expression patterns may explain the relatively modest increase in chymase levels observed upon stimulation with SGPs.

The mMCP-4 is the predominant connective tissue-type chymase, sharing substrate specificity, proteoglycan affinity, and tissue distribution with human chymase (Andersson et al. 2008). To explore the potential interaction of chymase with mosquito blood feeding, we conducted in vitro degradation assays using recombinant mMCP-6 and mMCP-4. Our data demonstrated that tryptase mMCP-6 showed minimal or no activity against these targets. In contrast, mMCP-4 selectively cleaved mosquito SGPs, including members of the D7 protein family, which are known to counteract host vasoconstriction and inflammation (Martin-Martin et al. 2023). These findings suggest that mMCP-4 could directly modulate the composition and functionality of mosquito saliva at the bite site, potentially impairing the vector’s ability to overcome host defenses.

Notably, while mast cell degranulation typically results in the release of proteases such as chymase that degrade foreign proteins, our finding, and those from prior studies, suggest that mosquito saliva may modulate this response to its own advantage. In our assays, *Ae. aegypti* SGPs induced mast cell degranulation and the release of mMCP-4, which we showed can potentially degrade salivary protein D7L1. Given that D7 proteins are essential for blood feeding due to their anti-hemostatic and anti-inflammatory properties, this might appear counterproductive. However, previous work has shown that certain mosquito salivary components can directly enhance the enzymatic activity of chymase. For example, *Ae. albopictus* salivary proteins 34k2 and adenosine deaminase were shown to significantly enhance human chymase activity (Li et al. 2022), suggesting an evolved mechanism by which mosquitoes may co-opt host chymase activity to promote vasodilation and facilitate blood meal acquisition. This interplay indicates that mosquito saliva may serve a dual function, triggering mast cell activation to enhance vascular permeability, while simultaneously modulating chymase activity to preserve or even enhance beneficial outcomes for the vector. Such a strategy may reflect evolutionary adaptation at the host–vector interface and warrants further investigation. This model may help explain the seemingly paradoxical observation that mosquito saliva triggers mast cell degranulation while also benefiting from the resulting protease activity.

Next, we investigated the role of mMCP-4 in *Ae. aegypti* blood meal intake using the Mcpt-4 knockout mice. We observed that the absence of chymase Mcpt-4 significantly delayed the blood feeding time and slowed blood feeding during the second bite. This indicates that in the absence of Mcpt-4, mosquitoes require more time to accomplish full engorgement. Chymase is known to induce the vasodilation, as it has been shown to cleave the tight junction proteins between the endothelial cells, recruit neutrophils and eosinophils, and increase of vascular permeability (Pejler 2020). All these can facilitate mosquito blood feeding. This might account for why some mosquito SPs could enhance the activities of chymase enzyme (Li et al. 2022). Together, these results highlight a previously underappreciated role for mMCP-4 in shaping host–mosquito interactions, both through immune modulation and direct degradation of key SPs involved in blood feeding.

The skin microbiome consists of diverse groups of commensal bacteria that play an important role in protecting against pathogens. However, when the microbiota dysbacteriosis occurs, it can contribute to inflammatory diseases by interacting with the immune cells residing in the skin (Belkaid and Segre 2014, Harris-Tryon and Grice 2022). MCs are essential in skin immunity by connecting the immune cells surrounded by them and linking the non-immune cells upon activation. MCs have the close connection with skin microbiota (Bosveld et al. 2023), for example, in atopic dermatitis, increased levels of the dominant skin bacteria *S. aureus* are highly related to the disease flare-ups (Kong et al. 2012). One mechanism is that the toxins released by *S. aureus* activated MCs, causing both innate and adaptive immune responses (Ashina et al. 2015).

In our study, we found that the mosquito bites induced very high abundance increase of 5 potential harmful bacteria Corynebacterium, Yaniella, *Lachnospiraceae_UCG-006*, Lachnoclostridium and *Candidatus_Saccharimonas* in Mcpt-4^fl/fl^ control mice. Of which, Corynebacterium is the relatively predominant bacteria which have been extensively studied in cutaneous disorders. Yaniella, *Lachnospiraceae_UCG-006* and Lachnoclostridium are potential harmful bacteria, and they have been extensively studied in gut-related diseases (Liang et al. 2020, Kan et al. 2022, Qu et al. 2024). The bacteria, *Candidatus_Saccharimonas*, occupy very low abundance in skin, with still limited known function. Importantly, MCs can be activated by aerobic *Corynebacterium equi* extract and be recruited to *Corynebacterium parvum* infected sites (Furuichi et al. 1982, Li et al. 1994). Additionally, Corynebacterium parvum was reported to have a capacity in enhancing the production of rabbit anti-BSA IgE (Pinckard and Halonen 1971). Additionally, Corynebacterium has been reported to increase the attractiveness of Anopheles Gambiae (Verhulst et al. 2011). In this study, we cannot prove whether Mcpt-4 expression affects blood feeding by modulating skin microbiota. However, based on our results, we hypothesize that the blood feeding process of mosquitoes may benefit from Corynebacterium, leading to enhanced mast cell degranulation and produce more IgE against SPs in order to release more vasodilation mediators, which can facilitate blood feeding.

In addition to the vasodilation function, chymase mMCP-4 plays immunomodulatory roles in many diseases. For instance, mMCP-4 affects the growth of Giardia infected mice by regulating intestinal cytokine expression (Li et al. 2020) and recruit leukocytes in the inflammatory phase of surgically wounded skin (Succar et al. 2019). During mosquito biting events, specific protease chymase mMCP-4, together with tryptase can be enhanced by SPs, suggesting the micro immune environment is influenced by the mMCP-4 regulation. This, in turn, may lead to alterations in skin microbiota through complex interaction.

## Conclusions

In this study, we discovered that, in vitro, the serum from mice bitten by *Ae. aegypti* mosquitoes promoted mast cell degranulation and increased chymase levels. Importantly, we found that recombinant mMCP-4 enzymatically degraded several *Ae. aegypti* SGPs, including D7 long-form salivary proteins that are critical for successful blood feeding. In vivo, our study revealed that blood feeding time was significantly delayed in the Mcpt-4 deficient mice. Absence of Mcpt-4 altered the skin’s bacterial composition and reduced abundance of Corynebacterium, Yaniella, *Lachnospiraceae_UCG-006*, Lachnoclostridium, and *Candidatus_Saccharimonas*, which are increased after mosquito biting in the WT mice. These findings highlight the crucial role of mast cell-derived chymase mMCP-4 in shaping mosquito blood-feeding and skin microbiota, which may influence the second biting behavior of *Ae. aegypti*.

## Limitations of the Study

While salivary gland proteins from *Ae. Aegypti* provide a relevant source for mast cell stimulation and are known to elicit antibody production in hosts, several limitations of the current study should be noted. One key limitation is that we did not quantify mast cell numbers in the skin of Mcpt-4-deficient mice. However, we consider it unlikely that Mcpt-4 deletion significantly altered mast cell density, as connective tissue-type mast cells are long-lived and typically persist for extended periods. Moreover, Mcpt-4 was deleted using a tamoxifen-inducible Cre-loxP system over a short time frame (5 d), which reduces the likelihood of substantial changes in mast cell populations. Nonetheless, histological quantification of mast cell numbers should be included in future studies to rule out any confounding effect. Additionally, while the mechanism by which Mcpt-4 deficiency reduces mosquito blood-feeding success remains incompletely understood, our current study did not directly assess vasodilation or inflammatory responses at the bite site, factors that could influence mosquito feeding efficiency. Instead, we evaluated differences in the cutaneous microbiota, supported by prior evidence that skin-resident microbes can shape local immune responses. In line with this, we observed altered microbiota composition and prolonged blood-feeding duration in Mcpt-4-deficient mice.

## Supplementary Material

tjaf137_Supplementary_Data

## Data Availability

Data from this study are available from the NCBI Sequence Read Archive: PRJNA1149461 (Li et al. 2025).
