# Supplementary material for: Mouse chymase mast cell protease-4 facilitates blood feeding of Aedes aegypti (Diptera: Culicidae) mosquitoes
Source: J Med Entomol. 2025 Oct 21;63(1):tjaf137. doi: 10.1093/jme/tjaf137 (PMC12823277; doi:10.1093/jme/tjaf137)
Supplement: tjaf137_Supplementary_Data [file tjaf137_supplementary_data.pptx]

## Slide 1
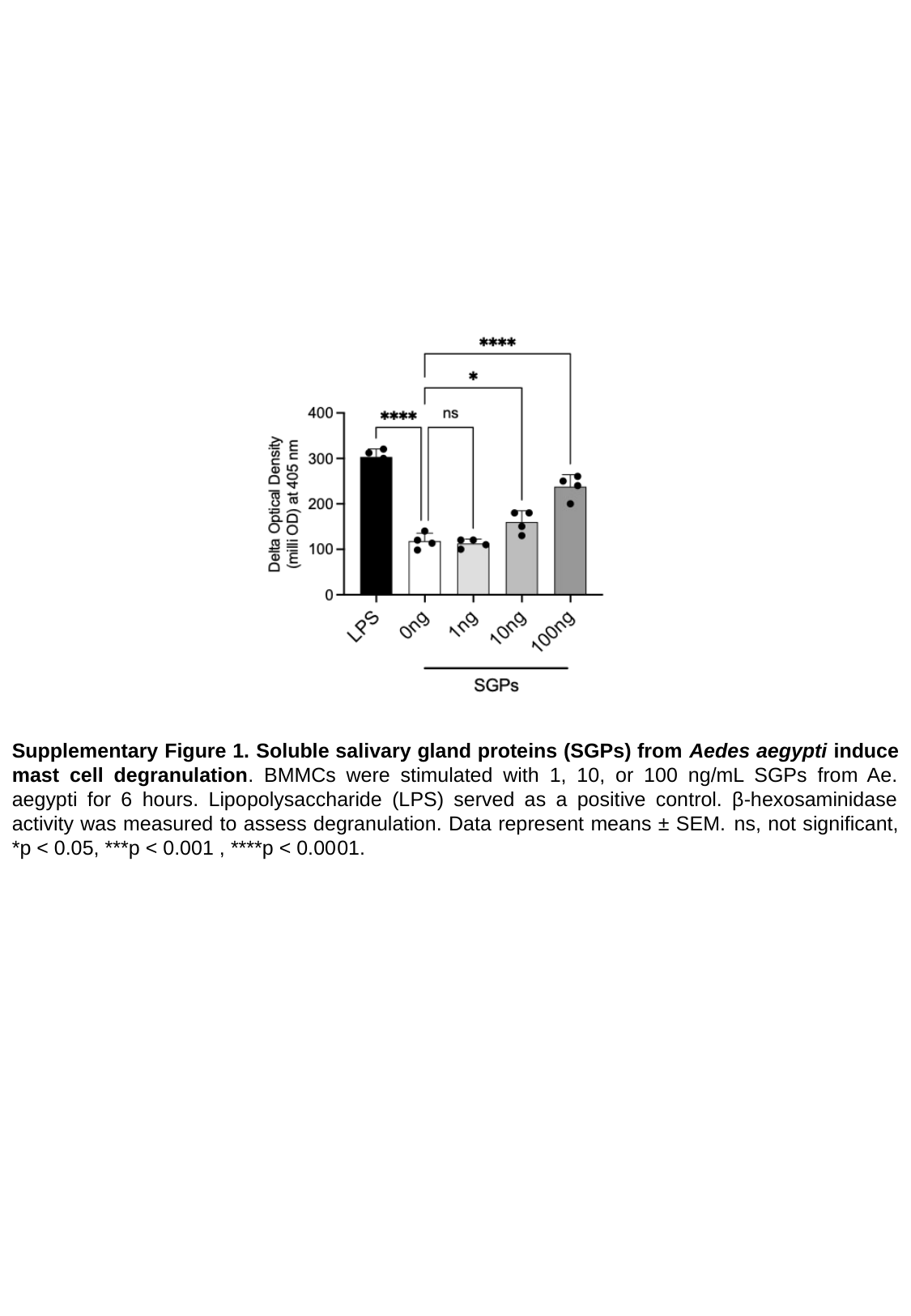

Supplementary Figure 1. Soluble salivary gland proteins (SGPs) from Aedes aegypti induce mast cell degranulation. BMMCs were stimulated with 1, 10, or 100 ng/mL SGPs from Ae. aegypti for 6 hours. Lipopolysaccharide (LPS) served as a positive control. β-hexosaminidase activity was measured to assess degranulation. Data represent means ± SEM. ns, not significant, *p < 0.05, ***p < 0.001 , ****p < 0.0001.

## Slide 2
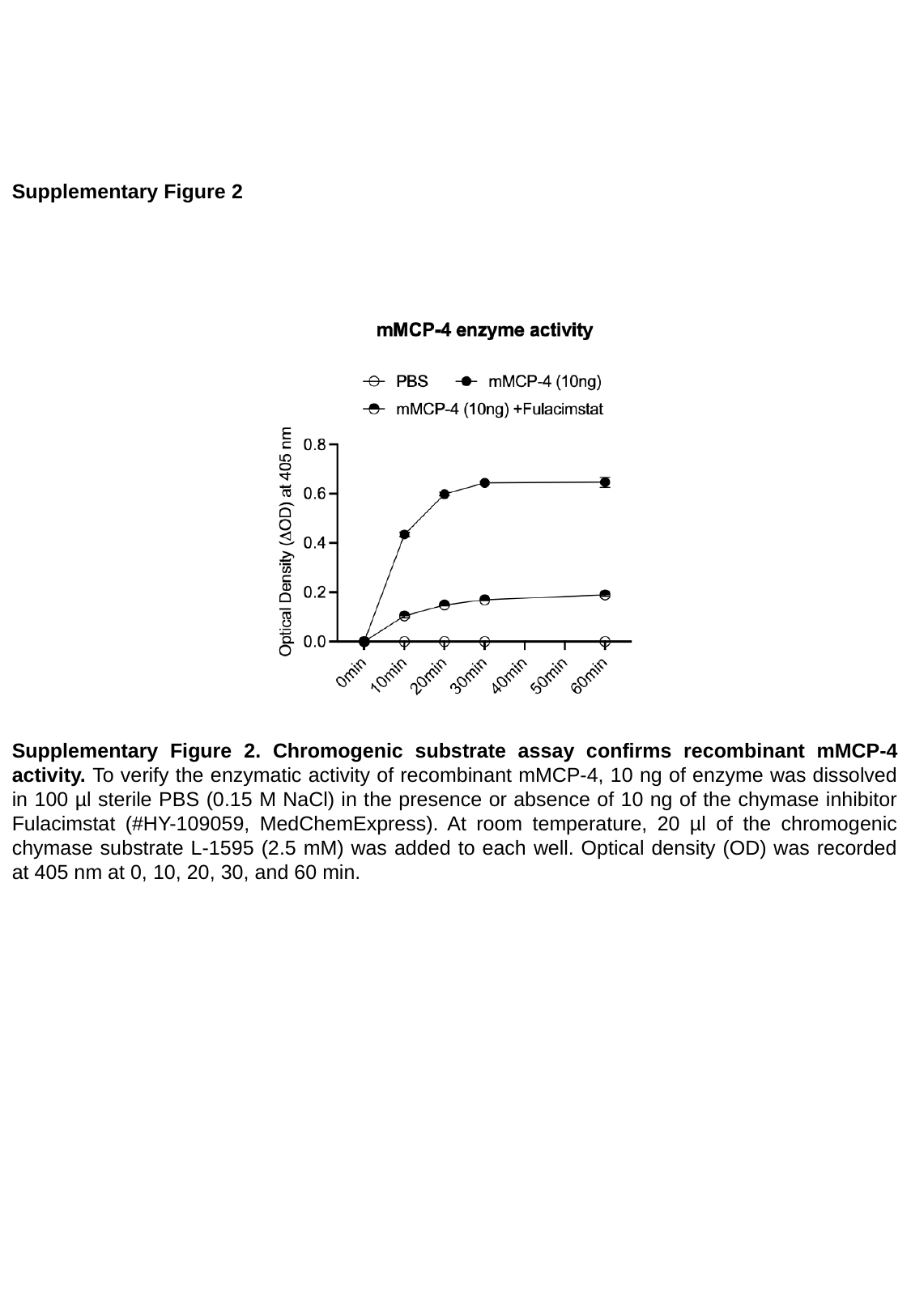

Supplementary Figure 2
Supplementary Figure 2. Chromogenic substrate assay confirms recombinant mMCP-4 activity. To verify the enzymatic activity of recombinant mMCP-4, 10 ng of enzyme was dissolved in 100 µl sterile PBS (0.15 M NaCl) in the presence or absence of 10 ng of the chymase inhibitor Fulacimstat (#HY-109059, MedChemExpress). At room temperature, 20 µl of the chromogenic chymase substrate L-1595 (2.5 mM) was added to each well. Optical density (OD) was recorded at 405 nm at 0, 10, 20, 30, and 60 min.

## Slide 3
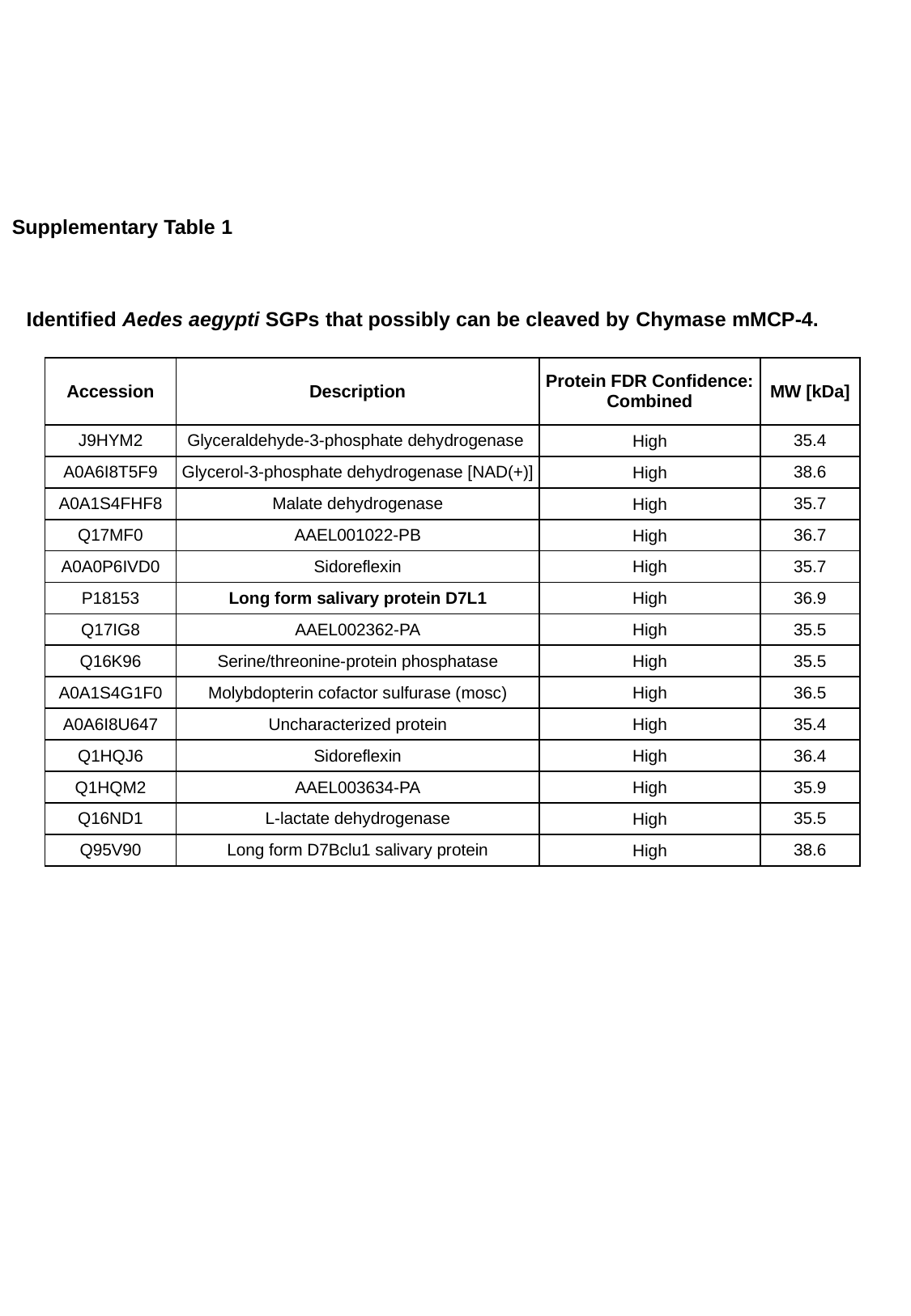

Supplementary Table 1
Identified Aedes aegypti SGPs that possibly can be cleaved by Chymase mMCP-4.
| Accession | Description | Protein FDR Confidence: Combined | MW [kDa] |
| --- | --- | --- | --- |
| J9HYM2 | Glyceraldehyde-3-phosphate dehydrogenase | High | 35.4 |
| A0A6I8T5F9 | Glycerol-3-phosphate dehydrogenase [NAD(+)] | High | 38.6 |
| A0A1S4FHF8 | Malate dehydrogenase | High | 35.7 |
| Q17MF0 | AAEL001022-PB | High | 36.7 |
| A0A0P6IVD0 | Sidoreflexin | High | 35.7 |
| P18153 | Long form salivary protein D7L1 | High | 36.9 |
| Q17IG8 | AAEL002362-PA | High | 35.5 |
| Q16K96 | Serine/threonine-protein phosphatase | High | 35.5 |
| A0A1S4G1F0 | Molybdopterin cofactor sulfurase (mosc) | High | 36.5 |
| A0A6I8U647 | Uncharacterized protein | High | 35.4 |
| Q1HQJ6 | Sidoreflexin | High | 36.4 |
| Q1HQM2 | AAEL003634-PA | High | 35.9 |
| Q16ND1 | L-lactate dehydrogenase | High | 35.5 |
| Q95V90 | Long form D7Bclu1 salivary protein | High | 38.6 |

## Slide 4
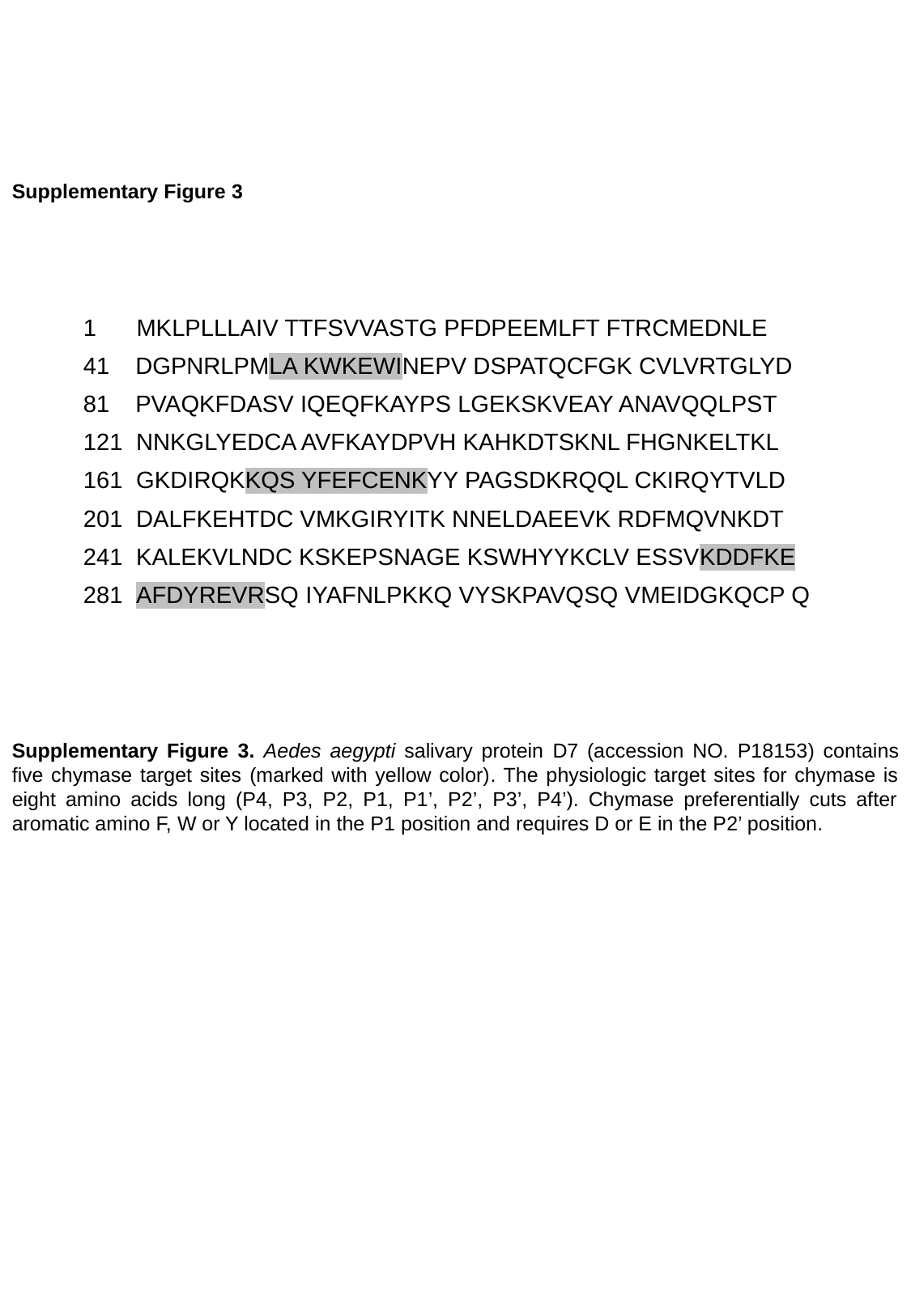

Supplementary Figure 3
1 MKLPLLLAIV TTFSVVASTG PFDPEEMLFT FTRCMEDNLE
 DGPNRLPMLA KWKEWINEPV DSPATQCFGK CVLVRTGLYD
 PVAQKFDASV IQEQFKAYPS LGEKSKVEAY ANAVQQLPST
121 NNKGLYEDCA AVFKAYDPVH KAHKDTSKNL FHGNKELTKL
161 GKDIRQKKQS YFEFCENKYY PAGSDKRQQL CKIRQYTVLD
201 DALFKEHTDC VMKGIRYITK NNELDAEEVK RDFMQVNKDT
241 KALEKVLNDC KSKEPSNAGE KSWHYYKCLV ESSVKDDFKE
281 AFDYREVRSQ IYAFNLPKKQ VYSKPAVQSQ VMEIDGKQCP Q
Supplementary Figure 3. Aedes aegypti salivary protein D7 (accession NO. P18153) contains five chymase target sites (marked with yellow color). The physiologic target sites for chymase is eight amino acids long (P4, P3, P2, P1, P1’, P2’, P3’, P4’). Chymase preferentially cuts after aromatic amino F, W or Y located in the P1 position and requires D or E in the P2’ position.
